# Mature cystic teratoma of the ovary: a cutting edge overview on imaging features

**DOI:** 10.1007/s13244-016-0539-9

**Published:** 2017-01-19

**Authors:** Hilal Sahin, Samir Abdullazade, Muzaffer Sanci

**Affiliations:** 1 Department of Radiology, Tepecik Training and Research Hospital, 35110 Yenisehir, Izmir Turkey; 2 Department of Pathology, Tepecik Training and Research Hospital, 35110 Yenisehir, Izmir Turkey; 3 Department of Gynecologic Oncology, Tepecik Training and Research Hospital, 35110 Yenisehir, Izmir Turkey

**Keywords:** Ovary, Cyst, Teratoma, Neoplasm, Dermoid

## Abstract

Mature cystic teratoma (MCT) is the most common neoplasm of the ovary and includes at least two well- differentiated germ cell layers. Different combinations of mature tissue derivatives with varying arrangements in the tumour cause a wide spectrum of radiological presentation ranging from a purely cystic mass to a complex cystic mass with a considerable solid component. In different imaging modalities, each radiological feature reflects a specific pathologic equivalent that forms because of diverse compositions of histological components. Understanding uncommon findings as well as the classic signs with basic knowledge of pathological equivalents permits a more accurate diagnosis and guides adequate treatment. In this review, radiological features of MCT in different imaging modalities (US, CT, MR imaging) including specific signs and useful radiological artefacts with brief emphasis on pathological basics are discussed.

*Teaching points*

• *Ovarian mature cystic teratomas (MCTs) have a wide spectrum of radiological presentation*.

• *Each radiological feature of MCT reflects a specific pathologic equivalent*.

• *Understanding radiological signs with basic knowledge of pathology can permit a more accurate diagnosis*.

## Introduction

Teratoma is the most common germ cell tumour of the ovary and accounts for about 20% of all ovarian neoplasms [1]. Pathologically, these tumours include a wide variety of germ cell origins, making the tumour pluripotent [2]. Ovarian teratomas are divided into sub-categories as follows: mature cystic teratomas, immature teratomas, monodermal (highly specialised) teratomas (struma ovarii, carcinoid tumours, neuroectodermal tumours, sebaceous tumours) and fetiform teratomas [2, 3]. Mature cystic teratoma (MCT) is the most common lesion of these tumours. Histologically, it includes at least two well-differentiated, so-called mature germ cell layers (ectoderm, mesoderm, endoderm). The ectoderm and mesoderm are the most commonly seen germ cell layers in the tumour wall; therefore, mature tissues of the skin and hair (from the ectoderm) and fat and muscle (from the mesoderm) usually make up the composition of MCT [2].

In imaging, MCTs have a wide spectrum of radiological presentation ranging from a purely cystic mass to a complex cystic mass with a considerable solid component. Ovarian teratomas are usually incidentally detected as a heterogeneous mass via ultrasonography (US). Although the fat component seems hyperechogeneous, it is not always easy to differentiate it from a complex haemorrhagic cyst. However, some radiologic signs, such as the dot-dash and tip of the iceberg signs, enable radiologists to diagnose teratomas sonographically. Cross-sectional imaging methods (computed tomography and magnetic resonance imaging) have excellent sensitivity for the diagnosis of MCT due to identification of fat [4]. In addition to detection of fat, other clues such as the fat-fluid level, floating ball sign, palm tree-like protrusion and reversed chemical shift artefact may be seen. Minute quantities of fat can also be detected with chemical shift imaging (in/opposed phase). These radiologic findings help radiologists to evoke the correct diagnosis of MCT. The incidence and appearance of each morphological feature and sign of ovarian teratomas in different imaging modalities are summarised in Table 1.

**Table 1:** Incidence and appearance of morphological features and signs of ovarian teratomas in different imaging modalities

Imaging feature/ sign	Incidence	US	CT	MRI
Rokitansky nodule (dermoid plug)	81–86% [5, 6]	Shadowing echodensity: a densely echogenic tubercule projecting into the cystic lumen	A rounded structure protruding into to the cystic lumen, mural thickening, a bridge across the cyst, a cystic structure or sometimes only tooth	A rounded structure protruding into to the cystic lumen, mural thickening, a bridge across the cyst or a cystic structure
Tip of the iceberg sign	4% [7]	Mixture of fatty fluid, hair and cellular debris creating an echogenic focus with acoustic shadowing behind it		
Dot-dash sign	61% [5]	Hyperechoic lines and dots arising from hairs in different orientations within the imaging plane		
Fat-fluid / fluid-fluid level	8–12% [5, 6]	Anechoic sebum layered above hyperechoic aqueous/debris containing layer or less frequently, supernatant hyperechoic sebum layer above hypoechoic aqueous fluid	Supernatant fatty layer with lower attenuation and dependent fluid layer with higher attenuation	High SI of supernatant fatty layer on T1-W images and low SI on fat-suppressed T1-W images
Floating balls sign	Uncommon [4]	Intracystic floating hyperechoic globules moving with changing position of the patient	Floating globules in gravity- independent position within the cyst fluid or in the interface of fat- fluid level	Floating globules in gravity- independent position within the cyst fluid or in the interface of fat-fluid level
Comet tail appearance	12% [7]	Hypoechoic hair balls with posterior acoustic shadowing		
Intratumoral fat	93% [6]	Diffuse or regional high amplitude echoes	A component with density between −144 and −20 HU in Rokitansky nodule or cyst wall, a layering component or a floating mass mixed with hair	A component with high SI on T1-W images and signal drop on fat-saturated T1-W images
Tooth/ calcification	56% [6]	Regional high amplitude echoes with shadowing	Curvilinear or globular calcification in the Rokitansky nodule, in the tumour wall or in/near the septa	
Chemical shift artefact	86% [8]			Foci or areas of very high SI on T2-W images or a boundary artefact with high and low SI bands on opposite sides of the tumour
Tuft of hair	65% [6]	Diffuse or regional high amplitude echoes		A component with chemical shift artefact in the gravity-dependent part of the cyst
Palm tree-like protrusion	21% [8]			Polipoid mass protruding into cyst cavity with internal pattern resembling a palm tree
Intratumoral keratinoid material	75% [9]			A component with low SI on T1-W and high SI on T2–W images and diffusion restriction

CT, computed tomography; MRI, magnetic resonance imaging; SI, signal intensity; US, ultrasound; W, weighted

This review covers common and uncommon imaging features, pathognomonic signs and useful radiologic artefacts in the diagnosis of MCT with a brief discussion of pathological equivalents.

## Clinical features

MCTs are seen in a younger age group (mean age 30 years) than epithelial ovarian neoplasms [10]. They are seen bilaterally in 12% of the cases [11]. In unilateral cases, MCT occurs more frequently on the right side (72.2%) [12].

Most MCTs are benign and asymptomatic unless a complication or a paraneoplastic syndrome develops. They grow slowly at a rate of 1.8 mm/year [13]. They are usually large at the time of diagnosis and often detected incidentally at routine pelvic examination [14]. On the other hand, they can be associated with various complications such as torsion (16% of ovarian teratomas), rupture (1%–4%), malignant transformation (1%-2%), infection (1%) and autoimmune haemolytic anaemia (<1%) [15]. In the case of complications, patients are managed in a different way; therefore, immediate and accurate diagnosis is important.

## Histopathological features

MCT usually looks like a unilocular cystic cavity at macroscopic examination. Also it may include septa dividing the cyst into several compartments. The tumour cavity is filled with sebaceous material because of the squamous epithelia in the wall. This material is liquid at body temperature and semisolid at room temperature [16]. There is usually a raised protuberance, known as the Rokitansky protuberance, projecting into the cyst cavity [2]. Bone and teeth tend to locate in this protuberance if they are present [14]. Also, most of the hair arises from this nodule as well as floating together with keratin and sebum in the lumen. Fat is present in more than 93% of the cases [8]. Sebaceous liquid material contains most of the lipid content whereas adipose tissue is less common [4].

Histologically, mature tissues from different cell lines lie within the wall. Walls of the cyst are frequently lined by squamous epithelium and often hyalinized, compressed ovarian stroma covers the external surface [10]. In an MCT, ectodermal elements are almost always present. When ectodermal tissues predominate, these teratomas are called as dermoid cysts [14]. Endodermal tissues (mucinous or ciliated epithelium) can also be seen in the majority of cases and mesodermal tissues are present over 90% of cases [4].

The presence of any immature tissue warrants a diagnosis of immature teratoma. If the teratoma is predominantly or exclusively composed of one germ cell line (endodermal or ectodermal), then it is referred to as a monodermal teratoma [14].

## Imaging features

### Ultrasonographic features

Ultrasonography is the most commonly used imaging modality for assessment of pelvic genital organs. The major role of US is to confirm the presence of a mass and determine its organ of origin [4]. By assessment of the internal architecture and echogenicity of the ovarian mass, the differential diagnosis may be limited. However, echogenic, fluid-filled masses may occasionally simulate solid lesions and are encountered in the differential diagnosis of MCT [8].

MCTs may show a variety of appearances sonographically because of their variable composition. However, they usually have a non-specific appearance. The sonographic appearance of ovarian cystic teratoma has been described to have “virtually limitless combinations” of different echo patterns [17]. Therefore, the diagnosis of MCT may be difficult by sole use of US. Sonographically, MCTs may seem as purely or predominantly cystic, solid or a complex mass with high reflection and acoustic shadowing [4]. There are also some specific sonographic signs that allow the diagnosis of MCT. These sonographic signs refer to different echo patterns such as the “thin echo pat-tern” or “dense echo pattern” or their combination [4]. When two or more characteristic signs are present, the diagnosis of MCT can be made with a high positive predictive value [5].

#### Shadowing echodensity (Rokitansky nodule or dermoid plug)

**Fig. 1:**
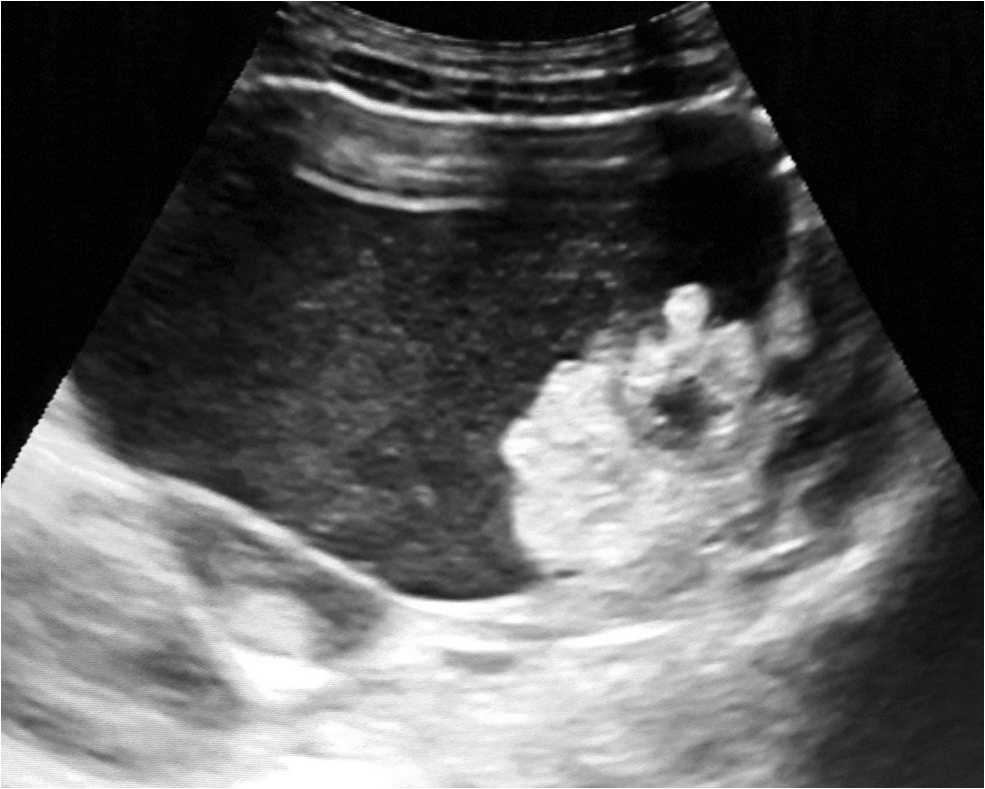
Transverse sonogram of a 20-year-old female with a mature cystic teratoma. A densely echogenic protuberance on the left side of the wall projecting into the cystic lumen is consistent with a Rokitansky nodule

**Fig. 2:**
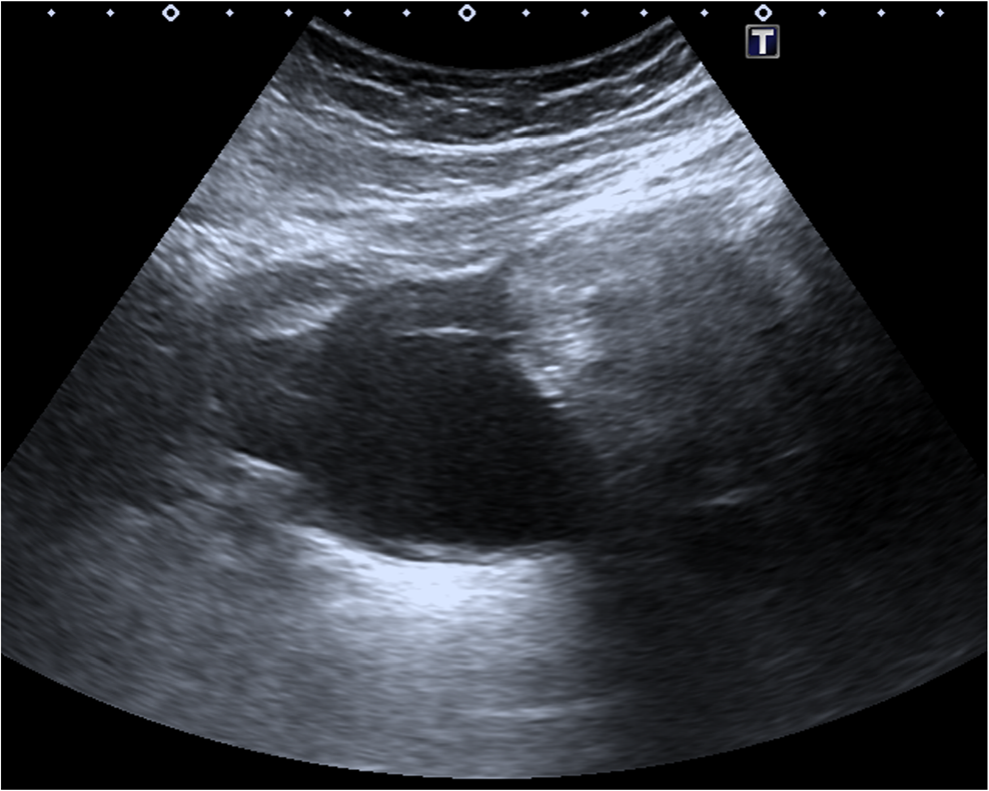
Transverse sonogram of an 18-year-old female with mature cystic teratoma. Shadowing echodensity projecting into the cystic lumen is seen, which was proved to contain fat and hair in the pathological specimen

**Fig. 3:**
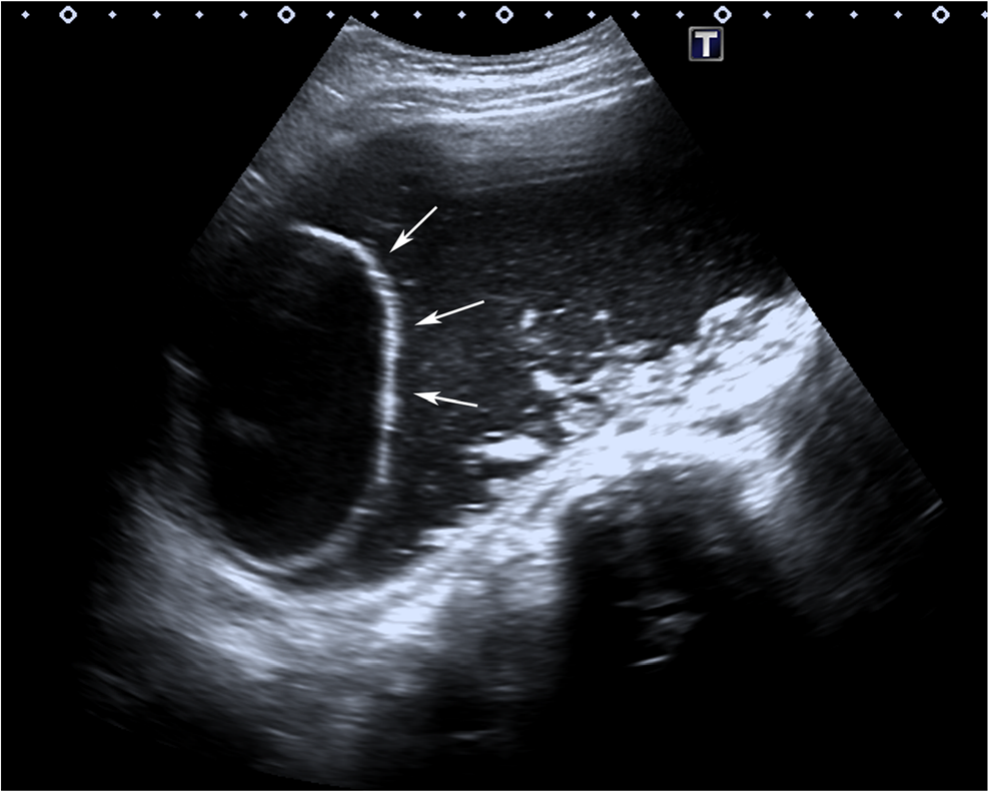
Transverse sonogram of a 14-year-old female with mature cystic teratoma demonstrates a cystic Rokitansky nodule (arrows) in a cystic mass. Note that there is echogenic debris at the gravity-dependent portion of the cyst

The Rokitansky nodule is the most common sonographic typical finding and is seen as a densely echogenic protuberance projecting into the cystic lumen (Fig. 1) [5]. This nodule, which is also called a dermoid plug, may show acoustic shadowing due to hair, teeth and the fat content (Fig. 2) [18]. Although it is described as a rounded solid mass, a bridge across the cyst or a thickened segment of the wall, it may rarely appear completely cystic (Fig. 3) [19, 20]. The Rokitansky nodule is a common site of malignant transformation and should be sectioned appropriately during pathologic analysis [1].

#### Diffuse or regional high-amplitude echoes

**Fig. 4:**
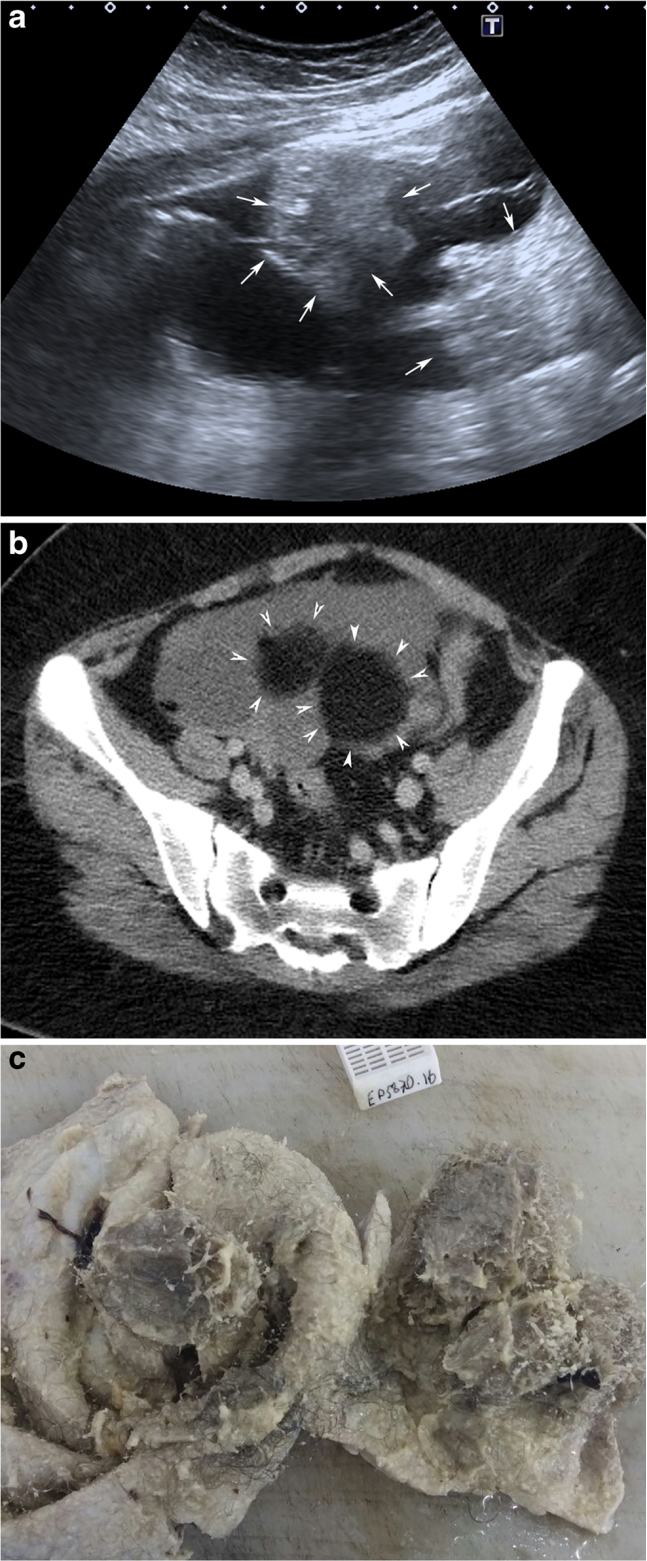
Transverse sonogram of a 16-year-old female with mature cystic teratoma (**a**) demonstrates high-amplitude echoes in two different regions (arrows). Axial computed tomography image (**b**) shows that those regions contain fat (arrowheads). In the macroscopic specimen (**c**), whitish sebaceous material with fat and hair is seen in the opened cystic lumen

This finding is the second common manifestation of MCT [5]. It results from the presence of mixed sebaceous material and hair [2]. The increased echogenicity may be diffuse throughout the mass or focal with accompanying sound attenuation (Fig. 4) [21]. In 8% of cases, diffuse or regional high-amplitude echoes may be seen without recognisable sound attenuation [5]. As a differential diagnosis, haemorrhage can also produce bright echoes. However, other distinctive features of the haemorrhage (such as fibrinous strands or a retracting clot) differentiate blood-containing masses from MCT [5].

#### Tip of the iceberg sign

**Fig. 5:**
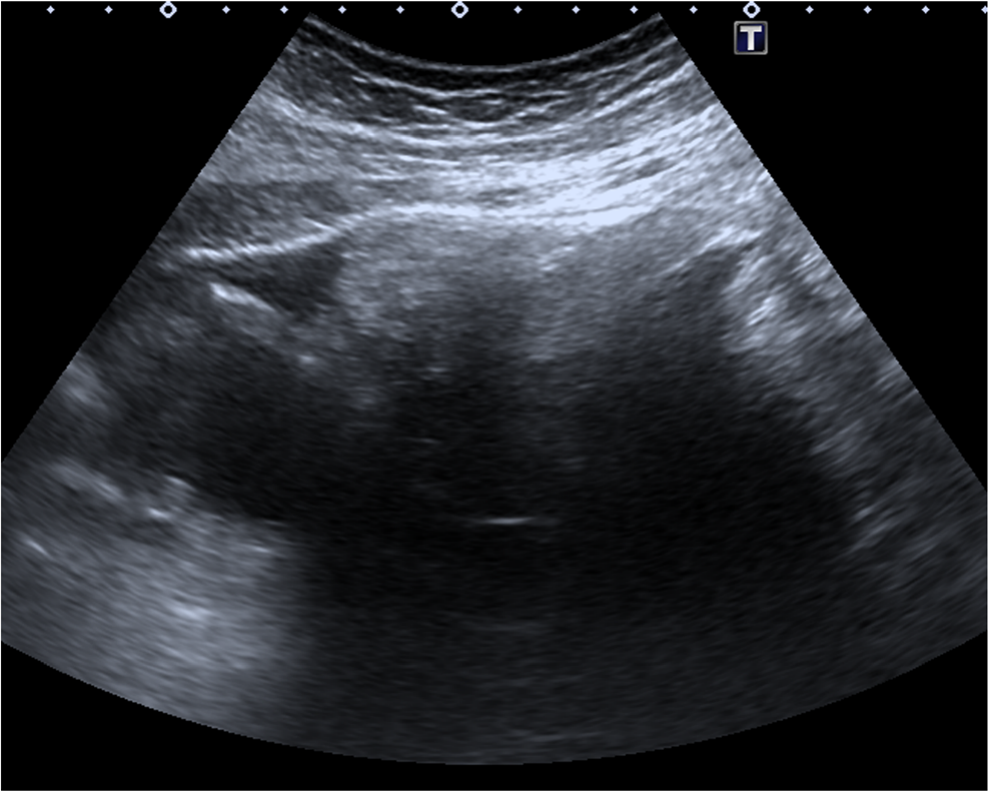
Transverse sonogram of a 16-year-old female with a mature cystic teratoma. An amorphous echogenic region is seen in the near field that causes posterior shadowing and obscures the posterior portion of the lesion and any structures behind it

This sign refers to the sonographic appearance of a mass with an amorphous echogenic focus in the near field that causes posterior shadowing and thus obscures the posterior portion of the lesion and any structures behind it (Fig. 5) [22]. This analogy to icebergs is appropriate in this instance because only a small volume of an iceberg (i.e. one tenth of the total volume) remains above the water [23]. Similarly, most of the volume of an MCT is obscured and invisible at US because of the strong posterior acoustic shadowing. The echogenic focus is actually a mixture of fatty liquid, matted hair and cellular debris, which can be identified on both transabdominal and transvaginal US scans [22]. The presence of highly reflective and attenuating hair within the sebaceous material and multiple tissue interfaces within this mixture produce the characteristic acoustic shadowing [22, 24]. This appearance is referred to as a characteristic sign of a dermoid cyst [22, 23].

In fact, acoustic shadowing is an attenuation error artefact that can be used by the clinician to determine the composition of a structure [25]. When the ultrasound beam encounters a strongly attenuating or highly reflective structure, which is the hair-sebaceous material mixture in MCT, the strength of the beam distal to this structure diminishes and becomes weaker than the beam in the surrounding field. This phenomenon is recognised as a shadow deep to highly attenuating structures [25].

As a diagnostic pitfall, the echogenic dermoid plug can resemble bowel gas or faecal material in the colon so that it can be easily overlooked or dismissed, particularly when sound attenuation obscures the posterior wall of the lesion creating the tip of the iceberg sign [26]. Therefore, when there is a palpable mass present clinically that is not seen on US, the examiner should look for an echogenic dermoid plug that was initially misinterpreted as bowel gas. As a clue, bowel gas and faecaloid material typically appear more reflective, with acoustic noise and ring-down artefact, than a dermoid plug, which attenuates sound more gradually over a greater depth [26]. In addition, an echogenic shadowing structure should be watched for evidence of peristalsis.

#### Dot-dash sign

**Fig. 6:**
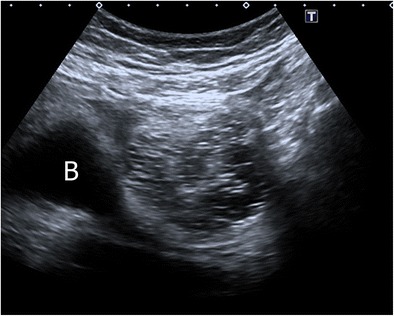
Transverse sonogram of a 51-year-old female with mature cystic teratoma. Echogenic lines and dots, making the dot-dash sign, are seen in a left adnexal mass regarding hair arranged in different orientations. *B* bladder

The classic sonographic appearance of echogenic hair floating within an MCT is described as the “dot and dash” sign, which has the highest positive predictive value (98%) [5]. This sonographic feature refers to hyperechoic lines and dots arising from hairs in different orientations within the imaging plane, which is also called the “dermoid mesh” sign (Fig. 6) [27]. These lines and dots metaphorically resemble the diagrammatic representation of International Morse Code characters, with “dots” representing echoes generated by hairs perpendicular to the scan plane and “dashes” representing echoes from hairs parallel to the scan plane [28]. Haemorrhagic cysts present a potential pitfall since fibrinous strands in a haemorrhagic cyst may mimic this appearance [5].

#### Fat-fluid or fluid-fluid level

Sebum floating above aqueous fluid forms a fat-fluid level separated by a linear interface that changes position with gravity. However, a layered appearance in a cyst on US is not specific for MCT. Fat-fluid or fluid-fluid levels have prognostic significance only when identified in combination with at least one additional sonographic feature associated with MCT [5].

**Fig. 7:**
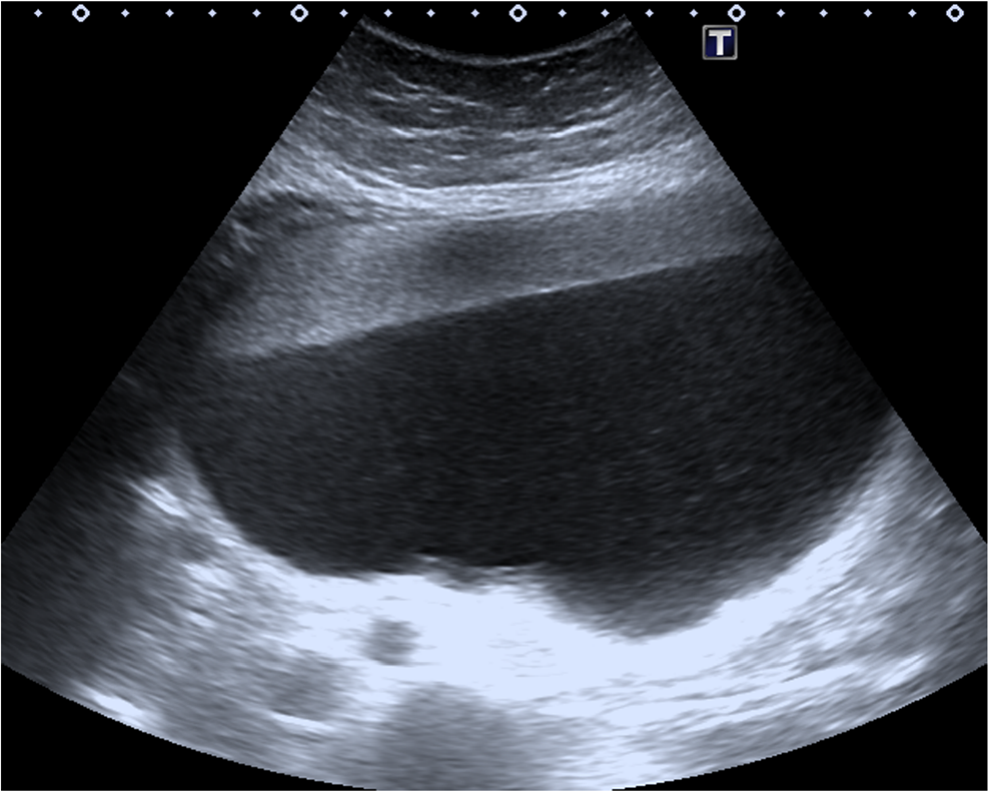
Transverse sonogram of an 18-year-old female with fat-fluid level in a mature cystic teratoma. Supernatant hyperechoic sebum forms a layer with dependent hypoechoic aqueous fluid

The histological composition of the fatty component, varying from pure liquid sebum to adipose tissue intermixed with hair, desquamated epithelium or fibrous tissue, results in different acoustic properties [29]. When there is pure sebum in the cyst, it may be observed as a hypoechoic or anechoic image [4]. Inclusion of water droplets due to the emulsifying role of the sebum and the presence of lipophilic contaminations, such as desquamated epithelial cells, give the sebum an echogenic appearance at US [30]. In addition, serous fluid may be anechoic or echogenic because of proteinaceous material or debris. The most common arrangement in this context is an anechoic sebum layered above a hyperechoic aqueous/debris-containing layer. Less frequently, supernatant hyperechoic sebum forms a layer with dependent hypoechoic aqueous fluid (Fig. 7) [21]. Also, a bright fluid interface may be seen with supernatant and dependent hypoechoic layers [29].

#### Floating balls sign

**Fig. 8:**
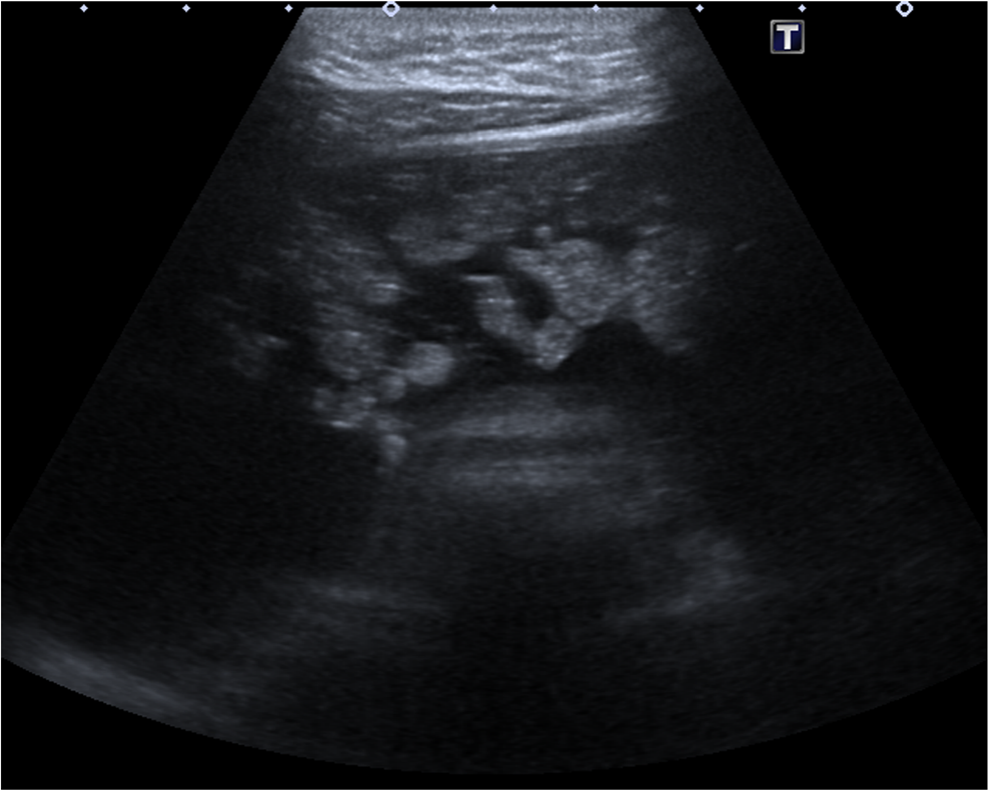
Transverse sonogram of a 14-year-old female with mature cystic teratoma shows multiple floating spherical echogenic structures that differ in size and shape

Intracystic hyperechoic floating balls are an uncommon pathognomonic feature of MCT (Fig. 8) [21]. In the literature, this sign, which is also called the “meat ball” sign, is described in few case reports or case series [31–33]. The size of the balls varies up to 4 cm and these globules are usually found in large MCTs. These balls are hyperechoic because of cheese-like sebaceous material, keratin and hair, with keratin being the major component [31, 34]. In addition, these globules float within the cyst because their specific gravity is equal to or less than the cyst fluid at body temperature. When examined dynamically with US, movement of these balls may be seen when changing the patient’s position [21]. Absence of blood flow in the Doppler US within these mobile spherules allows differentiation from solid mural nodules [31].

#### Comet tail appearance

**Fig. 9:**
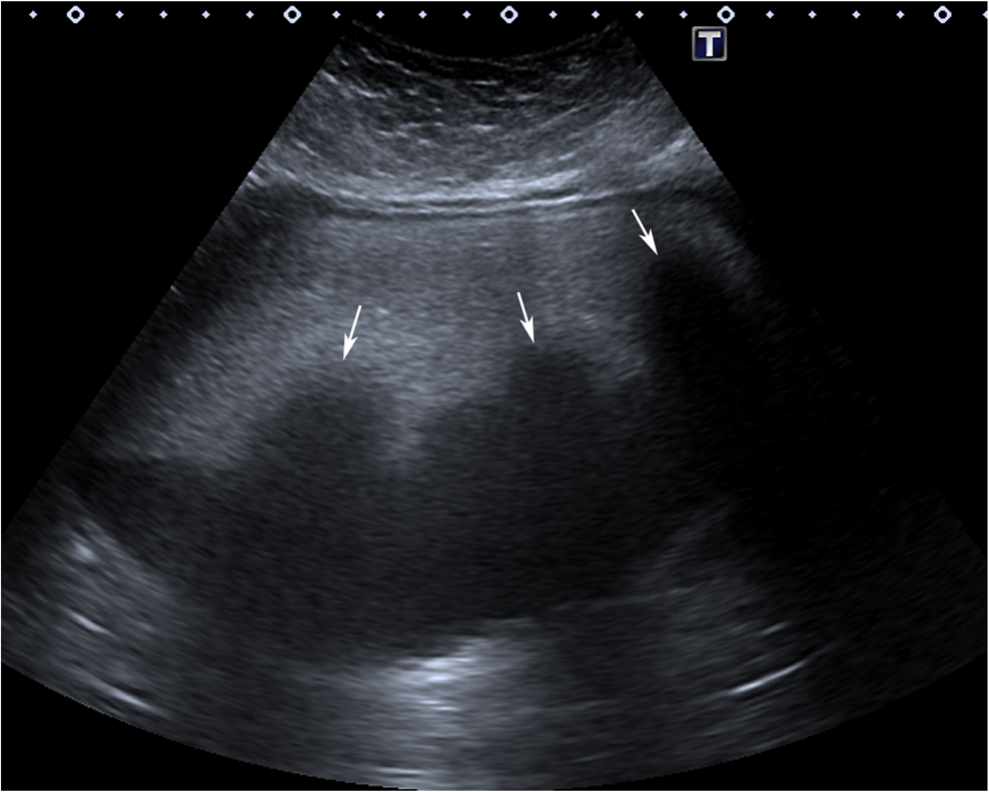
Transverse sonogram of an 18-year-old female with a mature cystic teratoma. There are three dark comet tail appearances (arrows) floating in the interface of the fat-fluid level consistent with hair balls pathologically

When hair makes a level with fluid, a comet tail appearance forms [7]. Due to attenuation of the sound beam in tufts of hair, a dark comet tail appears behind it (Fig. 9). This sign is different from a sonographic comet tail artefact, which is a form of reverberation [25]. The comet tail appearance is actually an acoustic shadowing, a form of attenuation error artefact, which is similar to the “tip of the iceberg” sign. However, in the comet tail appearance, an echogenic focus is not seen on the tip. In addition, hair balls create this appearance, which does not seen hyperechogenic as in the floating balls sign, and their posterior borders are not seen because of shadowing.

### CT imaging features

CT has an excellent sensitivity (93–98%) because of detecting fat in the diagnosis of MCT [4]. When US is nondiagnostic, CT examination of a teratoma is helpful in both demonstrating fat and subtle calcifications, in addition to delineating the effect of the mass on surrounding structures [19].

#### Intratumoral fat

**Fig. 10:**
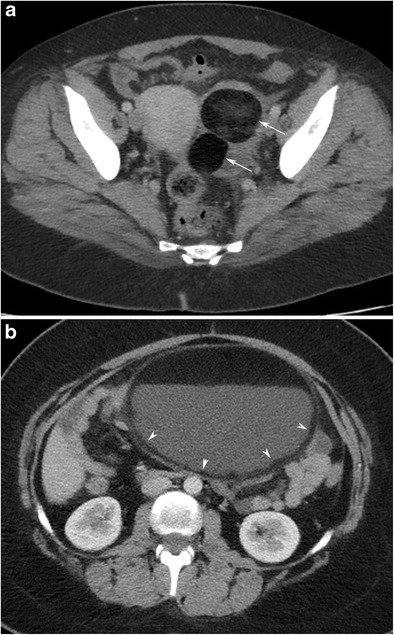
Axial CT image of a 25-year-old female with mature cystic teratoma (MCT). Two round fatty structures (arrows) are seen in the left adnexal mass (**a**). In another case with the fat-fluid level in an MCT, the axial CT image (**b**) shows a thin fat layer covering the inner side of the wall (arrowheads)

The presence of fat inside an ovarian tumour appears to be specific to ovarian cystic teratoma [6]. Fat may be seen as a round mass floating in the interface between two liquid components, a layering component, a floating mass intermingled with hair or a component in the cyst wall or Rokitansky nodule (Fig. 10) [6].

Fat displays lower attenuation than water on CT images; therefore when the proportion of fat within a voxel is large enough, the corresponding image pixel seems dark, with a density number ranging from −144 to −20 HU [4]. Although fat is simply identified by CT when it is macroscopic, detecting fat may be difficult when it is in minute quantities. On the other hand, mixing of sebaceous material with hair and debris increases the density of fat and, due to the water density of this mixture, fat may be overlooked [35].

#### The Rokitansky nodule (dermoid plug)

**Fig. 11:**
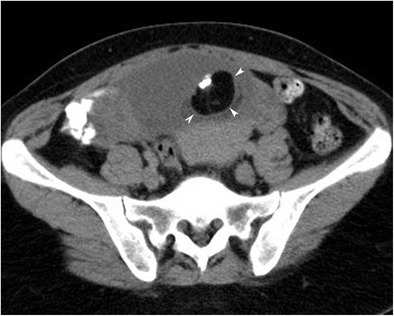
Axial CT image of a 37-year-old female with a mature cystic teratoma shows a rounded Rokitansky nodule (arrowheads) made up of fat and a tooth-like structure in a high-density cystic mass

On the CT image, a Rokitansky nodule can look like a rounded structure protruding into the cystic lumen, mural thickening, a bridge across the cyst or sometimes only a tooth (Fig. 11) [35]. The Rokitansky nodule is important because of the possibility of malign transformation. Contrast enhancement of a Rokitansky nodule should raise the possibility of malignant transformation, which does not always indicate malignancy [36]. In addition to an enhancing nodular soft tissue component, an obtuse angle between the soft tissue and the inner wall of the cyst, and extracapsular tumour growth with extension into an adjacent structure should be carefully examined for differentiation of malign from benign teratoma [36].

#### Tooth/calcification

**Fig. 12:**
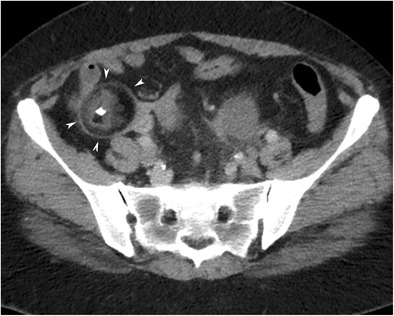
Axial CT image of an 18-year-old female with mature cystic teratoma shows a tooth-like high-density structure in the centre of a Rokitansky nodule (arrowheads)

It is possible to see curvilinear or globular calcification in the Rokitansky protuberance, in the wall of the MCT or in/near the septa (Fig. 12) [6]. In general practice, suggestive patterns of MCT include a fatty mass with curvilinear calcification and water density mass containing solid tissue components and globular calcification [19]. Calcification with very high HU values (higher than bone) suggests the presence of enamel and a tooth-like structure. When the ovarian MCT does not include fat or calcification, CT may not be helpful in the differential diagnosis [19].

#### Fat-fluid level

Two different liquid components can be clearly demonstrated by measuring their attenuation values on CT. Supernatant fatty layers of lower attenuation, similar to that of subcutaneous fat, and dependent fluid layers of higher attenuation are classically seen on CT (Fig. 10b) [29]. Sometimes, a floating nodule appearance may be seen with a fat-fluid level, which is accepted as pathognomonic for cystic teratoma [29, 37].

#### Floating balls sign

**Fig. 13:**
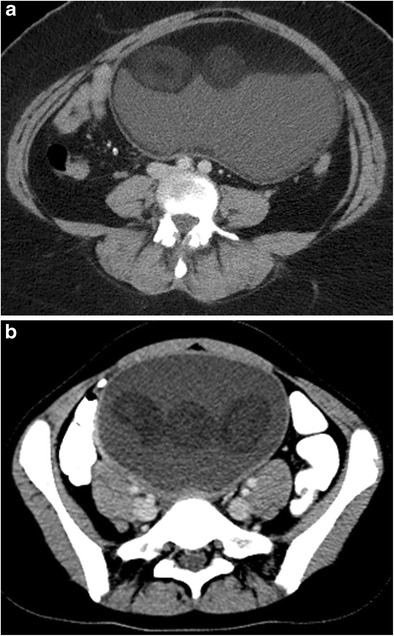
Axial CT image of an 18-year-old female with mature cystic teratoma (MCT) (**a**) shows two rounded floating balls in the interface of the fat-fluid level. The bigger one has a low density core and a low density outer shell. In another case with MCT (**b**), floating balls have relatively high density cores

A striking uncommon finding of MCT is numerous small spherical structures within the cyst (Fig. 13) [31]. Usually, these are discrete, uniformly sized mobile globules. The reason for the uniformity in the size of the globules is not known, but it is thought that each globule forms by the aggregation of sebaceous material around a tiny focus of debris, some desquamative material or fine hair shafts [38]. Differences in the physical and thermal properties of the material being deposited around each nidus create discrete masses rather than an amorphous mass [38]. Furthermore, the specific gravity of the globules is low because of fatty composition; therefore, they float in the most gravity-independent position within the cyst fluid or in the interface when there is a fat-fluid level [33]. Some spherules may have a 2–3-mm-thick outer sebaceous shell and a less dense central core [31]. In addition, peripheral low density of the floating balls on CT occurs as a result of decreased attenuation of the x-ray beam as it passes through the outer sebaceous/fat shell [33].

### MR imaging features

#### Intratumoral fat

**Fig. 14:**
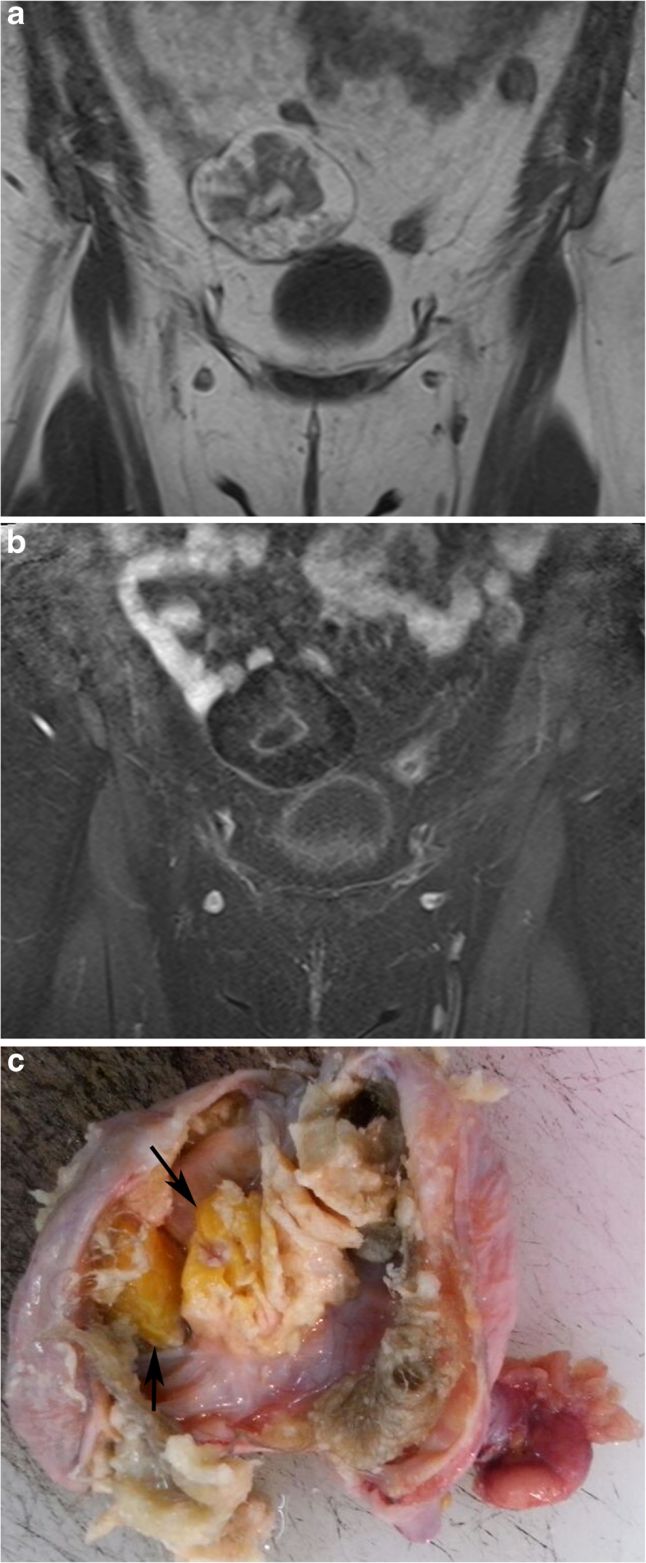
Coronal T1-weighted MR image of a 40-year-old female with mature cystic teratoma (**a**) shows a heterogeneous mass with high signal intensity areas in the right adnexa. In the fat-saturated T1-weighted image (**b**), the major fat component of the lesion is suppressed regarding the diagnosis of teratoma. In the macroscopic specimen (**c**), the fat component is seen as a yellow area at the centre of the tumour (arrows)

**Fig. 15:**
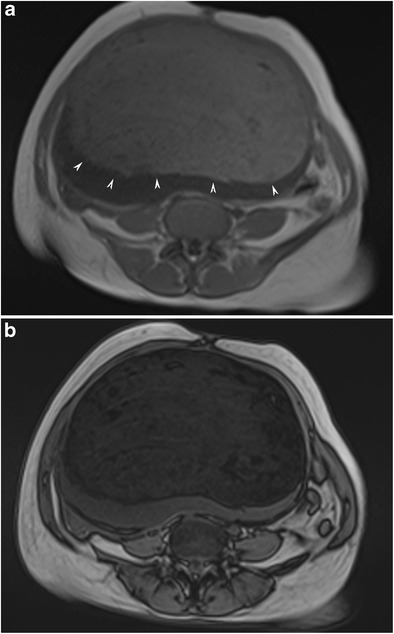
Axial T1-weighted in-phase MR image of a 56-year-old female with mature cystic teratoma (**a**) shows a large mass with a high signal intensity part creating an interface (arrowheads) with a low signal intensity part. T1-weighted opposed-phase image (**b**) demonstrates the fat component with decreased signal intensity in the supernatant layer regarding the presence of sebum

On MR imaging, intratumoral fat shows high intensity on T1-weighted images and a signal drop on fat-saturated T1-weighted images (Fig. 14) [15]. Since tissues with a short T1 value (i.e. endometrioma) can display signal characteristics similar to those of fat, fat suppression sequences are an indispensable adjunct to standard sequences in gynaecological MR imaging [39]. The most widely used method to distinguish fat from blood is the frequency selective fat saturation technique [40]. Also chemical shift imaging (in/opposed phase, Dixon methods) is commonly used and is the most sensitive technique to detect minor fat (Fig. 15) [4, 39]. On the other hand, the STIR (short TI inversion recovery) sequence is not chemical shift-specific; therefore, it should be used with non-fat-saturated T2-weighted images because low signal intensity on STIR suggests either a suppressed fatty component or haemorrhage [2]. MR imaging may fail to visualise very small fatty areas (mean diameter about 1 mm) when compared with CT, which can be explained by a better signal/noise ratio in CT, the thinner CT slices and possibly magnetic field inhomogeneities in the boundary of the calcified structures [39].

#### Chemical shift artefact

**Fig. 16:**
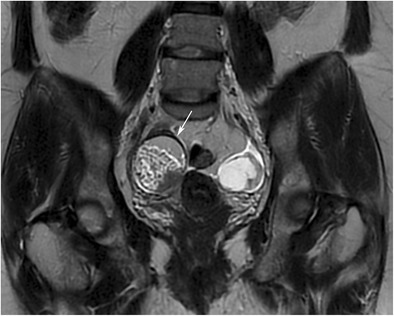
Coronal T2-weighted MR image of a 42-year-old female with mature cystic teratoma shows chemical shift artefact both inside the tumour and in the borders. A low signal intensity band (arrow) is seen on the cranial border of the cyst with a high intensity band on the opposite side

Teratomas may show reversed chemical shift artefact, which is a boundary artefact perpendicular to the direction of the frequency encoding gradient [8]. This artefact can be used to detect fat and distinguish it from haemorrhage [2]. It is seen as foci or areas of very high signal intensity in T2-weighted images at the interfaces of fatty tissue and non-fatty tissue, and a low-intensity band is seen on the opposite side of the tumour (Fig. 16). This atypical artefact may be seen in and/or around the tumour and is characteristic in the diagnosis of ovarian teratomas [8].

#### Fat-fluid level

**Fig. 17:**
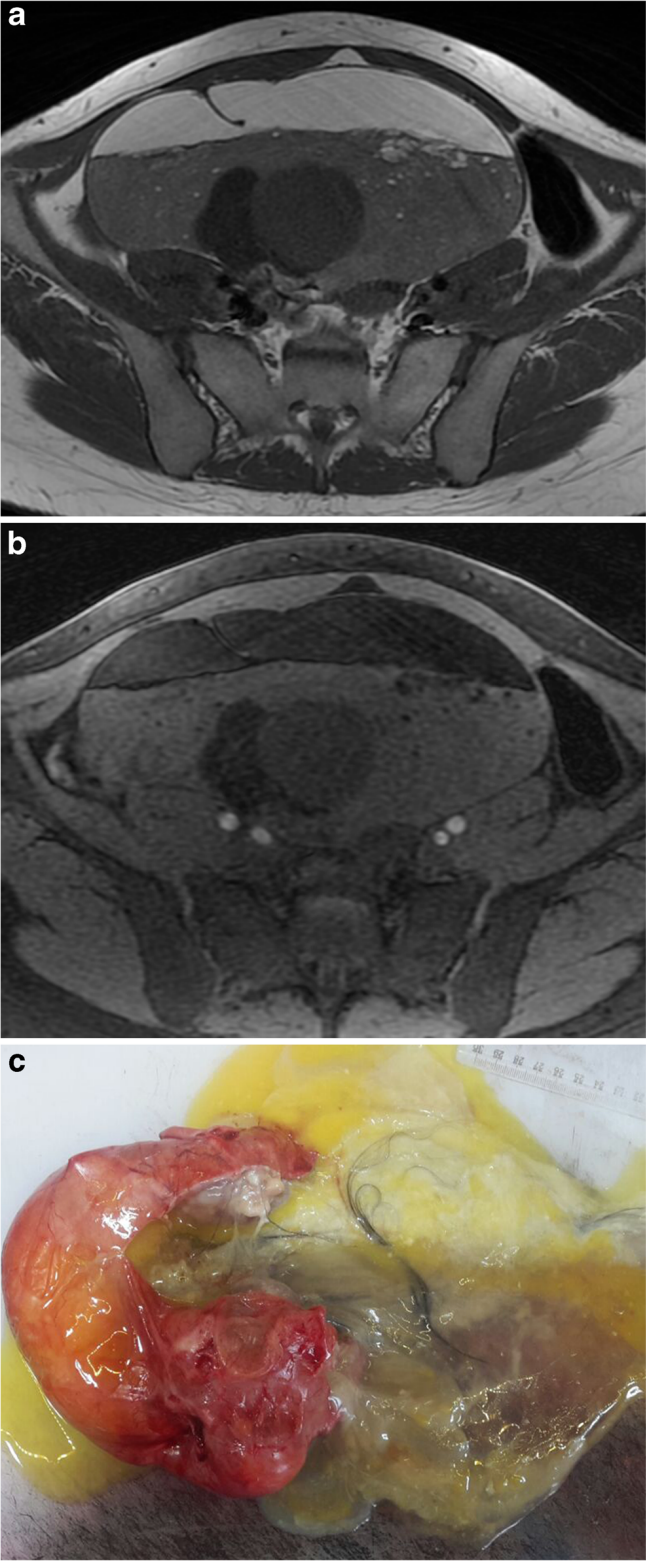
Axial T1-weighted MR image of a 31-year-old female with mature cystic teratoma (**a**) shows high signal intensity of the supernatant fatty layer creating an interface with a low signal intensity aqueous layer. Fat-saturated T1-weighted image (**b**) demonstrates a major fat component with decreased signal intensity in the supernatant layer and free-floating hypointense fat particles in the aqueous layer. A Rokitansky nodule with cystic components is seen in the posterior part of the cyst. Cut section of the tumour (**c**) shows yellowish sebaceous material leaking from the cystic lumen. Note the black hairs in that material

**Fig. 18:**
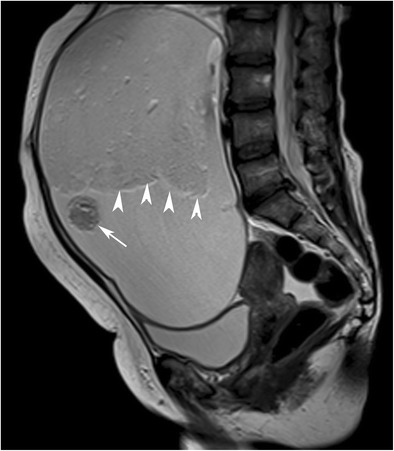
Sagittal T2-weighted MR image of a 56-year-old female (same case as in Fig. 15) with mature cystic teratoma shows chemical shift artefact (arrowheads) in the interface of two different hyperintense layers. Note that the complete gravity-dependent layering of the fatty component in the cranial part is not formed regarding the high viscosity. A floating rounded structure is also seen in the cystic lumen (arrow)

**Fig. 19:**
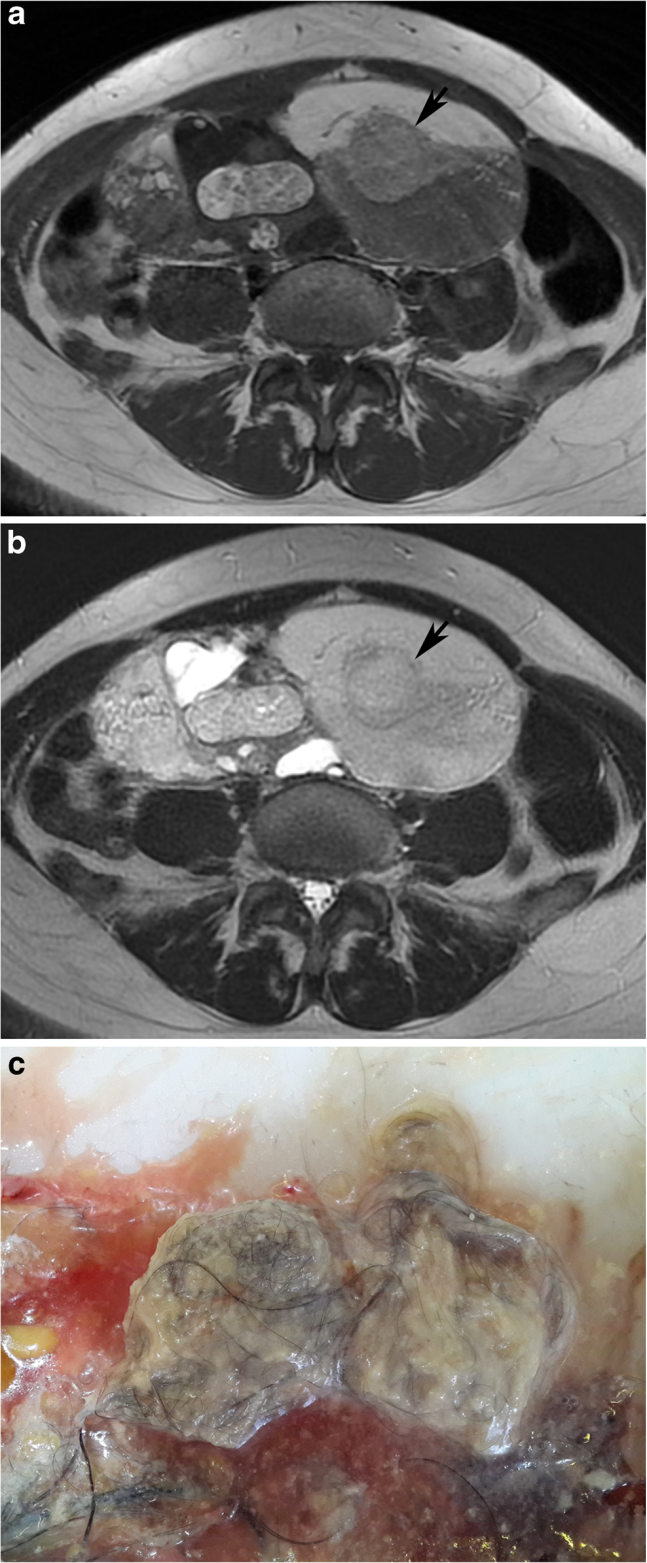
Axial T1-weighted MR image of a 31-year-old female with mature cystic teratoma (**a**) (same case as in Fig. 17). A floating ball is seen in the interface of the high signal intensity fat and low signal intensity aqueous fluid layer (arrow). Axial T2-weighted image (**b**) shows chemical shift artefact in and around the ball (arrow). In the macroscopic specimen (**c**), the floating ball corresponds to whitish creamy material containing hair and keratin

On MRI, fat and fluid may show layering with high signal intensity of the supernatant fatty layer on T1-weighted images and low signal intensity on fat-suppressed T1-weighted images (Fig. 17). On T2-weighted images, the supernatant and dependent layers show variable signal intensities [29]. Complete layering as a fat-fluid level may not be seen if the viscosity of the fatty fluid is high (Fig. 18). Rarely, one single ball made up of debris, fat and hair may be seen floating in the interface of the fat-fluid level (Fig. 19) [37].

#### The Rokitansky nodule (dermoid plug)

**Fig. 20:**
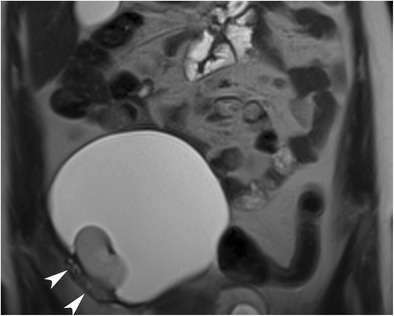
Coronal T2-weighted MR image of a 21-year-old female with mature cystic teratoma. A bean-shaped Rokitansky nodule is seen lying on the right side of the wall of the cystic mass. Ovarian parenchyma with normal signal intensity (arrowheads) can be seen near the cyst wall where the Rokitansky nodule arises

**Fig. 21:**
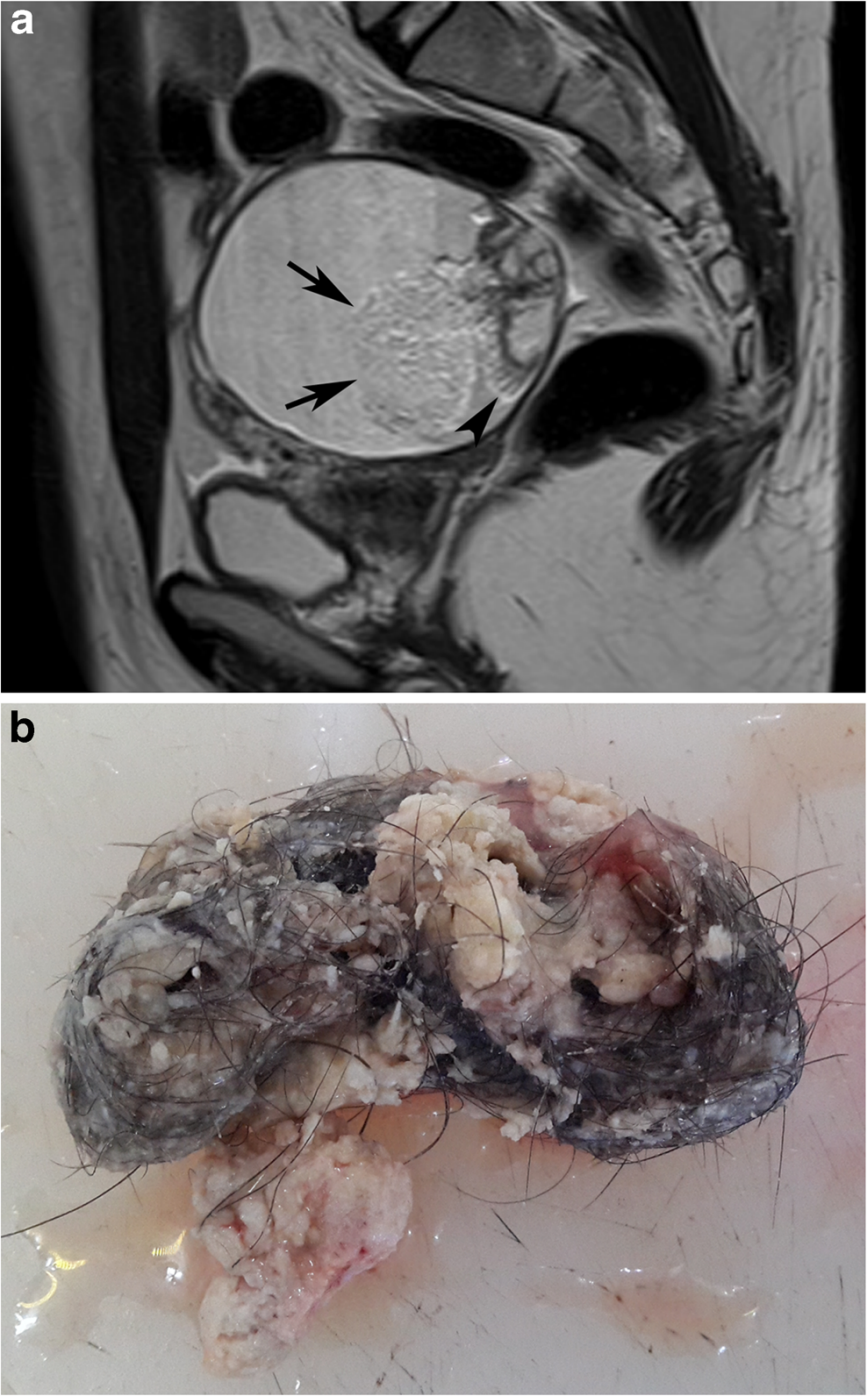
Sagittal T2-weighted MR image of a 22-year-old female with mature cystic teratoma (**a**). A hyperintense structure (arrows) with an obvious chemical shift artefact lying in the interface of the fat-fluid level is seen in a cystic mass. It is consistent with matted hair and keratinoid material in the macroscopic specimen (**b**). Note the Rokitansky nodule at the posterior wall with hyperintense parts that were proven to be fat and linear hair shafts (arrowhead) arising from the nodule in the T2-weighted image. Cut-section photograph of the Rokitansky nodule is shown in Fig. 25d

Morphological features of the Rokitansky nodule (i.e. size, number, relationship with the cyst wall, shape and content) can easily be evaluated with MRI (Figs. 20 and 21). On dynamic contrast-enhanced MR imaging, Rokitansky nodules of benign MCTs display variable enhancement patterns, which may correlate with the specific content of solid tissue in the nodule [41].

#### Tuft of hair

Matted hair, which usually constitutes the major part of the layered debris, is gravity dependent and may show mobility when changing the patient’s position (Fig. 21) [4]. This tuft of hair is usually mixed with whitish, cheese-like material and pronounced on MR images because of chemical shift artefact. With increasing amounts of hair, the intensity becomes lower on T2-weighted images [8].

#### Palm tree-like protrusion

**Fig. 22:**
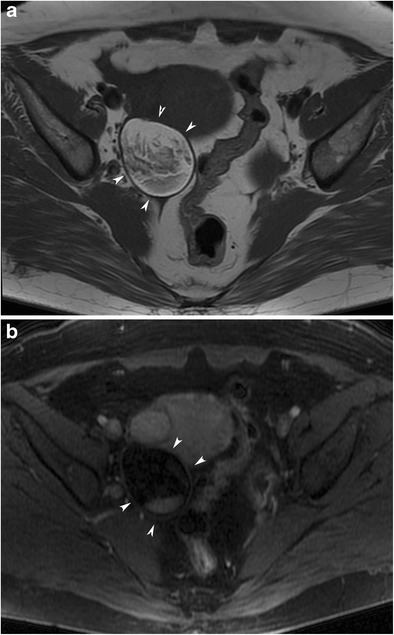
Axial T1-weighted MR image of a 42-year-old female with mature cystic teratoma (**a**) shows a palm tree-like structure projecting into the high signal intensity mass (arrowheads). Fat-saturated contrast-enhanced T1-weighted image (**b**) demonstrates a suppressed fat component in the tumour (arrowheads)

Protrusions, which are usually located at the point of contact with residual ovarian tissue, vary from small nodules to polipoid palm tree-like masses projecting into the cyst cavity (Fig. 22) [8]. The internal pattern resembling a palm tree is typical for teratomas [42]. The palm tree-like protrusion consists predominantly of desquamated, degenerated epithelial cells mixed with hair and the signal intensity varies according to the proportion of hair and cheese-like material [8].

#### Floating balls sign

**Fig. 23:**
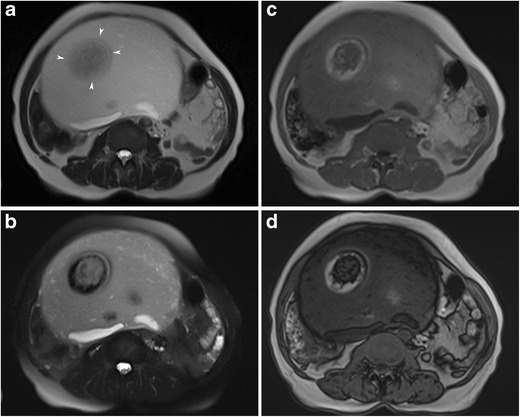
Axial T2-weighted (**a**), fat-saturated T2-weighted (**b**), in-phase (**c**) and opposed-phase T1-weighted (**d**) MR images of a 56-year-old female with a floating ball (arrowheads) in a mature cystic teratoma. The central core of the ball contains minor fat, which is seen as decreased signal intensity in the opposed-phase image, and an outer layer with a major fat signal that decreases in the fat-saturated T2-weighted image

Floating globules usually have a nidus composed of debris, desquamative material or fine hair shafts that is hyperintense on T2-weighted and hypointense on T1-weighted images [4]. The outer portion, which is formed by aggregation of sebaceous material around the nidus, has the opposite signal intensity compared to the inner part. These spherules may also contain macroscopic or microscopic fat, which can be detected by fat-suppression techniques or in phase-opposed phase imaging (Fig. 23). Sometimes floating small fatty particles, which are important for the diagnosis of teratomas without a major fat component, may also be seen without a nidus.

#### Intratumoral keratinoid material

**Fig. 24:**
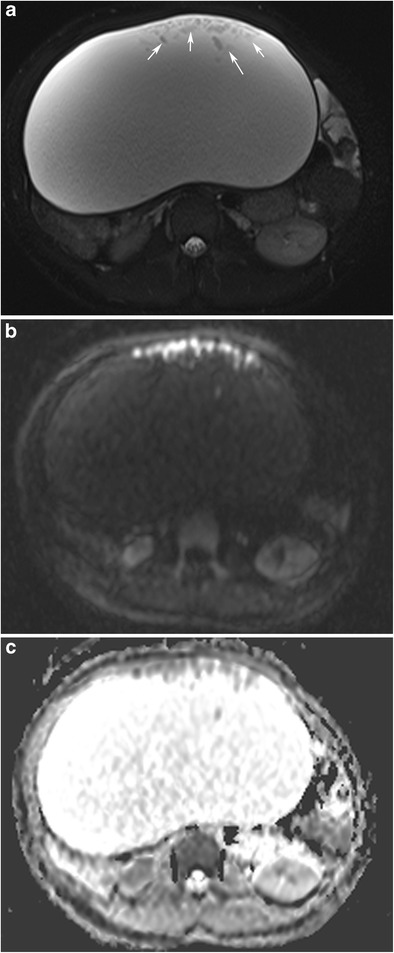
Axial fat-saturated T2-weighted (**a**), diffusion-weighted (*b 800*) image (**b**) and ADC map (**c**) images of a 14-year-old female with a mature cystic teratoma. Multiple floating structures (arrows) are seen in the anterior part of the cystic mass. Those structures show diffusion restriction regarding the keratin content, which was also proven pathologically

**Fig. 25:**
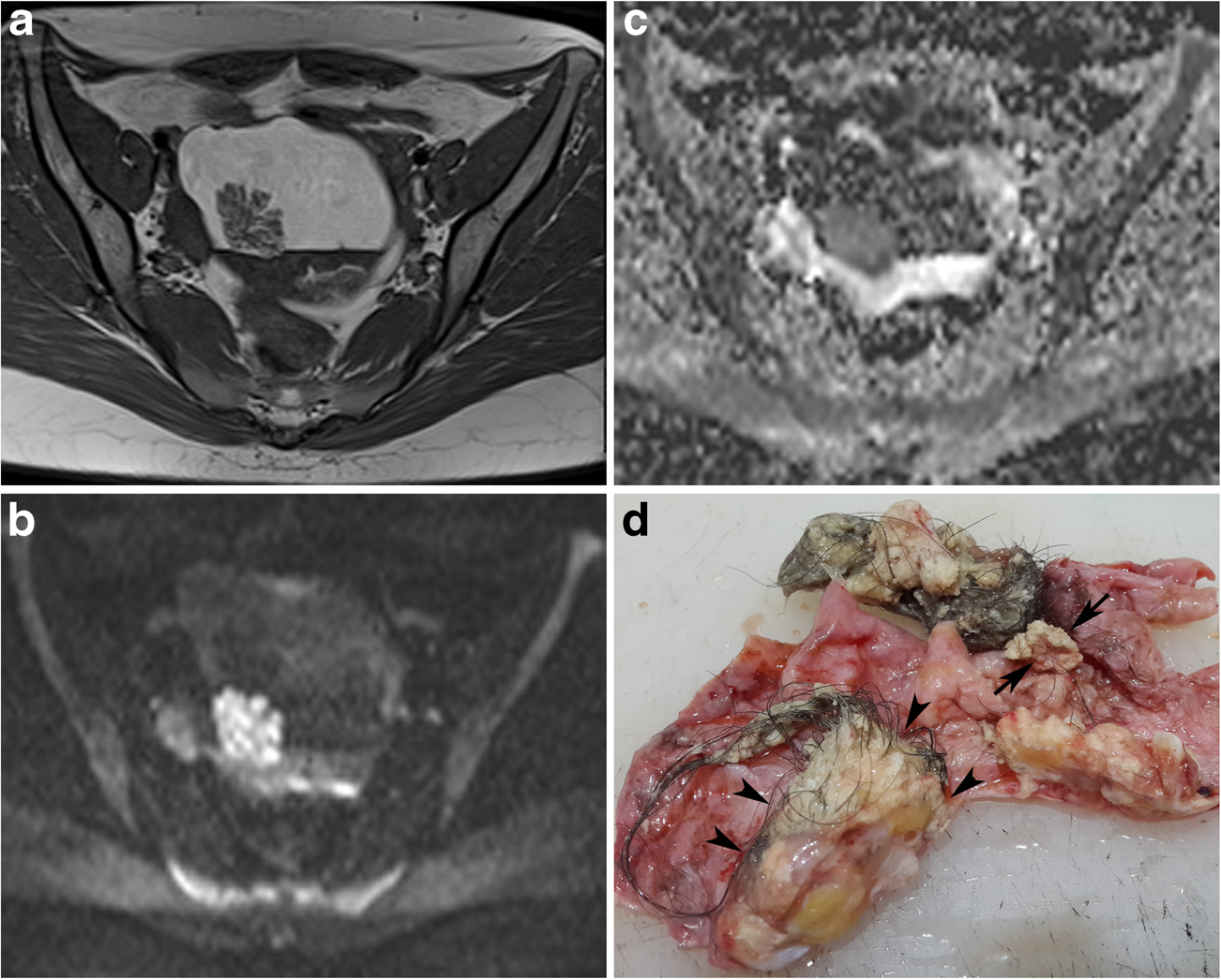
Axial T1-weighted (**a**), diffusion-weighted (*b 800*) image (**b**) and ADC map (**c**) images of a 22-year-old female with a mature cystic teratoma (same case as in Fig. 21). A hypointense cauliflower-like mass is seen near the interface of the fat-fluid level and a Rokitansky nodule is seen in the posterior part of the cystic lesion (**a**). The cauliflower-like mass shows diffusion restriction regarding the keratin content (**b**, **c**). Whitish cheese-like material regarding keratin (arrows) is seen in the lumen in the macroscopic specimen of the tumour (**d**). Cut section of the Rokitansky nodule (arrowheads) shows a yellowish fat component, creamy keratin and fine hair shafts arising from the nodule

The keratinoid substance is a scleroprotein that originates from the cytoskeletal structure of the epidermis. Radiologically it exhibits low signal on T1- and high signal on T2-weighted images resembling serous fluid [9]. However, diffusion- weighted imaging (DWI) can distinctly separate these two substances. Restriction of Brownian movement of water molecules within the keratinoid substance results in a high signal on DWI and a low ADC value (Figs. 24 and 25) [9]. Therefore, in a limited population of MCTs, particularly when fat is not obvious, DWI may serve as an adjunct tool for the correct diagnosis [43].

## Conclusion

MCTs have a wide spectrum of appearances in different imaging modalities. Each radiological sign is a description of a specific pathologic appearance and reflect the different combinations of histological components. Understanding uncommon findings as well as classic signs with basic knowledge of pathological equivalents permits a more accurate diagnosis and guide adequate treatment.

## Abbreviations

CT: Computed tomography
DWI: Diffusion-weighted imaging
US: Ultrasonography
MCT: Mature cystic teratoma
MR: Magnetic resonance
